# Clinical and Molecular Characterization of Brazilian Patients Suspected to Have Lynch Syndrome

**DOI:** 10.1371/journal.pone.0139753

**Published:** 2015-10-05

**Authors:** Felipe Carneiro da Silva, José Roberto de Oliveira Ferreira, Giovana Tardin Torrezan, Márcia Cristina Pena Figueiredo, Érika Maria Monteiro Santos, Wilson Toshihiko Nakagawa, Rafael Canfield Brianese, Ligia Petrolini de Oliveira, Maria Dirlei Begnani, Samuel Aguiar-Junior, Benedito Mauro Rossi, Fábio de Oliveira Ferreira, Dirce Maria Carraro

**Affiliations:** 1 Laboratory of Genomics and Molecular Biology, International Research Center/CIPE—A. C. Camargo Cancer Center, São Paulo, Brazil; 2 Department of Colorectal Tumors, A. C. Camargo Cancer Center, São Paulo, Brazil; 3 Anatomic Pathology Department, AC Camargo Cancer Center, São Paulo, Brazil; 4 National Institute of Science and Technology in Oncogenomics (INCITO), Brazil; Sapporo Medical University, JAPAN

## Abstract

Lynch syndrome (LS) accounts for 3–5% of all colorectal cancers (CRC) and is inherited in an autosomal dominant fashion. This syndrome is characterized by early CRC onset, high incidence of tumors in the ascending colon, excess of synchronous/metachronous tumors and extra-colonic tumors. Nowadays, LS is regarded of patients who carry deleterious germline mutations in one of the five mismatch repair genes (MMR), mostly in *MLH1* and *MSH2*, but also in *MSH6*, *PMS1* and *PMS2*. To comprehensively characterize 116 Brazilian patients suspected for LS, we assessed the frequency of germline mutations in the three minor genes *MSH6*, *PMS1* and *PMS2* in 82 patients negative for point mutations in *MLH1* and *MSH2*. We also assessed large genomic rearrangements by MLPA for detecting copy number variations (CNVs) in *MLH1*, *MSH2* and *MSH6* generating a broad characterization of MMR genes. The complete analysis of the five MMR genes revealed 45 carriers of pathogenic mutations, including 25 in *MSH2*, 15 in *MLH1*, four in *MSH6* and one in *PMS2*. Eleven novel pathogenic mutations (6 in *MSH2*, 4 in *MSH6* and one in *PMS2*), and 11 variants of unknown significance (VUS) were found. Mutations in the *MLH1* and *MSH2* genes represented 89% of all mutations (40/45), whereas the three MMR genes (*MSH6*, *PMS1* and *PMS2*) accounted for 11% (5/45). We also investigated the MLH1 p.Leu676Pro VUS located in the PMS2 interaction domain and our results revealed that this variant displayed no defective function in terms of cellular location and heterodimer interaction. Additionally, we assessed the tumor phenotype of a subset of patients and also the frequency of CRC and extra-colonic tumors in 2,365 individuals of the 116 families, generating the first comprehensive portrait of the genetic and clinical aspects of patients suspected of LS in a Brazilian cohort.

## Introduction

Lynch syndrome (LS), formerly known as Hereditary Non-Polyposis Colorectal Cancer (HNPCC), accounts for 3–5% of all colorectal cancers (CRC) and is inherited in an autosomal dominant fashion. This syndrome is characterized by early CRC onset, a high incidence of tumors in the ascending colon, an excess of synchronous and metachronous tumors, an accelerated adenoma-carcinoma transition (2–3 years) and an excess of extra-colonic manifestations (endometrium, small-bowel and ureter/renal pelvis cancers) [1].

LS was originally described as a familial clustering of CRCs according to the Amsterdam criteria I and II, based on three cases with CRC (or endometrial, small-bowel or ureter/renal pelvis); one being a first degree relative, and one of the relatives presenting with CRC before the age of 50; two generations must be affected, and Familial Adenomatous Polyposis should be excluded [2,3]. In 1997, it was developed the Bethesda guideline (BG) [4], which was revised in 2004 [5] to help identify patients at risk of LS based on tumor phenotype, e.g., tumors with genomic instability in repetitive sequences called microsatellites (MSI). CRC with MSI accounts for approximately 15% of all colorectal tumors and 90% of Lynch syndrome CRCs. Thus BG was proposed for both, identifying individuals at risk for LS and also recommending criteria for MSI testing.

Currently, LS is diagnosed in patients who carry germline mutations, including point mutations or genomic rearrangements, in one of the five Mismatch Repair (MMR) genes, i.e., MutL homolog 1 (*MLH1*), MutS homolog 2 (*MSH2*), MutS homolog 6 (*MSH6*), post-meiotic segregation increased 1 (*PMS1*) or post-meiotic segregation increased 2 (*PMS2*), [6–8]. Genetic testing for LS has been available for more than 20 years; however, it is not the standard care for all patients suspected of LS, and it is still restricted to the most frequently mutated genes, *MLH1* and *MSH2* [9–12]. It is noteworthy that the genetic diagnosis of LS patients is of great importance, because the early management and intensive surveillance programs can significantly reduce the incidence of LS-associated tumors and mortality rate for family members [13].

Mutations in the *MLH1* and *MSH2* genes account for 70–80% of all germline mutations detected in LS patients and mutations in *MSH6*, *PMS2* and *PMS1* genes are detected at lower frequencies. Altogether, *MSH6* and *PMS2* mutations account for 5–20% of kindreds negative for *MSH2* and *MLH1* mutations [14]. Mutations in both *MSH6* and *PMS2* genes have been suggested to affect families with atypical LS, which presents with a late onset of disease, lower incidence of colorectal cancer and a high incidence of endometrial cancer; the latter is noted for *MSH6* mutations only [15–20]. However, previous studies have demonstrated that the risk of cancers in patients with Lynch syndrome can vary across populations with distinct genetic backgrounds [21].

Recently, *EPCAM* germline deletion has also been associated to LS patients [22,23]. The chromosome localization of *EPCAM* gene is adjacent to *MSH2*. Deletion in the *EPCAM* leads to transcriptional read-through, which results in the silence of *MSH2* by hypermethylation [24]. Additionally, clinical data of *EPCAM*-deleted patients have shown that the extension of the deletions involving *MSH2* gene have different implications in colorectal cancer predisposition [25].

In this study, to comprehensively characterize Brazilian patients suspected of having LS, we generated a complete genetic depiction of the five MMR genes (*MLH1*, *MSH2*, *MSH6*, *PMS1* and PMS2) in a cohort of 116 patients. We performed germline mutations screening of *MSH6*, *PMS1* and *PMS2* in noncarriers of *MLH1* and *MSH2* point mutations who were previously described [12]. Moreover, we assessed chromosomal deletions/duplications in *MLH1*, *MSH2* and *MSH6* through Multiplex Ligation-dependent Probe Amplification (MLPA) and performed functional studies in one variant of uncertain significance (VUS) detected in the *MLH1* gene. Additionally, for a group of patients with no identified pathogenic mutations, we have performed MSI, MMR Immunohistochemistry and KRAS, NRAS and BRAF mutations. Finally, based on clinical and pathological data from all patients and their relatives, totaling 2,365 individuals, we determined the frequency of CRC and extra-colonic tumors in mutation-positive and negative families, providing, for the first time, an initial genetic and clinical description of families suspected of having LS within a cohort in Brazil.

## Materials and Methods

### Patients

The study used the Oncotree database of the Hereditary Colorectal Cancer Registry of the Pelvic Surgery Department of AC Camargo Cancer Center (São Paulo, Brazil). The analyzed families were selected according to the Amsterdam criteria I or II and the revised Bethesda guideline, which included patients with CRC diagnosed at age <50 year; presence of synchronous, metachronous CRC, or other LS associated tumors; CRC with the MSI-H histologyc diagnosed before 60 years old; CRC diagnosed in 1 or more first-degree relatives with a LS-related tumor, with one of the cancers being diagnosed before 50 years old; CRC diagnosed in 2 or more first- or second-degree relatives with LS-related tumors, regardless of age. All families were registered in the period between January 1998 and June 2009. The recruitment of the patients was from 2006 to 2009. One hundred sixteen unrelated families were eligible for this study. All patients signed an informed consent form. This study was performed in compliance with the Helsinki Declaration and was approved by the ethics committee of the A C Camargo Cancer Center under number 870/06.

### Clinical data

The cohort consisted of 2,365 individuals from 116 suspected LS families. The analysis included the number of CRC, extra-colonic tumors, number of evaluated generations and age at diagnosis. All tumors described in this study were based on verbal reports of the index patient or close relatives and confirmed by clinical or pathological data whenever possible.

### Screening of germline point mutations

Genomic DNA was extracted from peripheral blood in the macromolecule laboratory (AC Camargo Cancer Center Biobank), using the Puregene Genomic DNA Isolation Kit (Gentra Systems, USA) according to the manufacturer’s instructions.

The complete coding sequence of the five MMR genes and at least two bases corresponding to splice sites were screened for point mutations. For *MLH1* (NM_000249) and *MSH2* (NM_000251.1), PCR and sequencing protocols are described elsewhere [12]. Intronic primers were designed to evaluate coding exons of the *MSH6* (NM_000179.1) and *PMS1* genes (NM_000534.4). For *PMS2* (NM_000535.5) screening, long-range PCR was performed to avoid spurious amplification of pseudogenes (available upon request). All sequences were analyzed with CLC GenomicWorkbench software.

### Classification of the variants

The classification of the variants was based on the InSiGHT locus specific database (http://insight-group.org/variants/database/). All nonsense and frameshift alterations that should generate premature stop codons were classified as pathogenic. Missense alterations were evaluated using three protein prediction algorithms, namely, SIFT (http://sift.jcvi.org/), POLYPHEN–2 (http://genetics.bwh.harvard.edu/pph2/) and Align-GVGD (http://agvgd.iarc.fr/agvgd_input.php).

### MLPA (Multiplex Ligation-Dependent Probe Amplification) analysis

Large genomic rearrangements (LGR) involving the *MLH1*/*MSH2* and *MSH6* genes were investigated by MLPA (MRC-Holland, Amsterdam, The Netherlands) using the P003-B2 and P072 kits, respectively. Experiments were performed according to the manufacturer’s protocol. The analyses were performed with the Coffalyser v9 software.

### Target Sequencing for KRAS, NRAS and BRAF mutations, Microsatellite instability (MSI) and Immunohistochemistry (IHC)

For a group of patients with no MMR pathogenic mutations identified, their tumor tissues (fresh tissue or paraffin embedded tissue, available at the AC Camargo Cancer Center biobank) were investigated for point mutations in *KRAS* (codons 12, 31, 61, 117 and 146), *NRAS* (codons 12, 13, 61 an 146), *BRAF* (codon 600) and MSI. For these analysis, DNA was extracted from micro-dissected FFPE tumor sections using QIAamp DNA FFPE Tissue Kit (QIAGEN, Germany) and quantified using a Nanodrop (Thermo Scientific, Wilmington, DE).

Mutation analysis were carried out through massive parallel sequencing in Ion Torrent Personal Genome Machine platform using an Ion 316 Chip and Ion PGM Sequencing 200 Kit v2 (Thermo Scientific, Wilmington, DE). Reads were quality-filtered and sorted according to barcodes using Torrent Suite Browser 4.0.1, and Variant Caller plugin from Torrent Suite Browser was used for finding single nucleotide variants. The cutoff for classifying as mutated was 5% with a medium coverage of 500X.

MSI analysis was performed with the MSI Analysis System (Promega, Madison, Wisconsin, USA) consisting of mononucleotide repeats BAT–25, BAT–26, NR–21, NR–24, MONO–27, Penta C and Penta D in paired tumor-normal DNA samples, following manufacturer’s recommended amplification conditions for the MSI Analysis System. PCR products were denatured in deionized formamide with Internal Lane Standard 600 (Promega, Madison, Wisconsin, USA) for allele sizing and analyzed on a 3130*xl* Genetic Analyzer using GeneMapper 4.0 Software (Thermo Scientific, Wilmington, DE). Allelic sizes for matching normal and tumor samples were compared and considered MSI unstable if there was a shift of 3bp or more in the tumor allele. Samples were classified as MSI-High (MSI-H) when two or more markers out of seven were unstable, MSI-Low when one out of seven markers was unstable and MSI stable when there were no unstable markers.

Immunohistochemistry analysis of MLH1, MSH2, PMS2 and MSH6 proteins was performed in whole tissue slides stained with antibodies against MLH1 (clone G168-728, 1:600; BD Biosciences), PMS2 (clone A16-4, 1:500; BD Biosciences), MSH2 (clone FE11, 1:1000; BD Biosciences), MSH6 (clone 44, 1:40; BD Biosciences) using enzyme-conjugated polymer system. Expression of MLH1, MSH2, MSH6 and PMS2 were scored as positive when nuclear staining was observed in tumor cells and negative if the staining was observed in the internal control but not in the tumor cells.

### Target sequencing for patient ID–039

Seventeen hereditary cancer genes (*ATM*, *BRCA1*, *BRCA2*, *CDKN2A*, *CHEK2*, *CTNNB1*, *ECAD*, *FANCJ*, *MLH1*, *MSH2*, *MSH6*, *NBN*, *PALB2*, *PTEN*, *RAD50*, *RAD51* and *TP53*) were screened through target capture DNA sequencing using a custom HaloPlex Target Enrichment 1–500 kb design (Agilent, USA) according to the HaloPlex Target Enrichment System-Fast Protocol, Version B. This method was performed only for patient ID–039 for detecting germline mutation in other genes associated with hereditary cancer. The library was sequenced in an Ion PGM Sequencer using an Ion 316 Chip and the Ion PGM Sequencing 200 Kit v2 (Thermo Scientific, Wilmington, DE). The mean targeted base coverage depth was 400X. SNVs and indels were identified using the VariantCaller v4.0.r73742 plugin from the Torrent Suite Browser.

### Frequency of a Variant of Unknown Significance (MLH1 Leu676Pro) in the control population

For assessing the frequency of the MLH1 p.Leu676Pro variant in the Brazilian population, we tested a control cohort of 280 DNA samples from healthy subjects with no family history of cancer. Sequencing reactions were performed using the Big Dye v.3.1 cycle sequencing kit on an ABI Prism 3500 genetic analyzer (Thermo Scientific, Wilmington, DE).

### Functional assays

DNA plasmid construction and site-directed mutagenesis were carried out using pcDNA 3.1/myc-His A (Invitrogen), and the expression constructs for the missense variant was designed using the pcDNA MLH1 wild-type vector (hMLH1) and the QuikChange II-E Site-Directed Mutagenesis Kit (Agilent, Santa Clara, California, USA). Cell transfection was carried out using the colon cancer cell lines HCT–116 (ATCC CCL–247™) and SW–480 (CCL–228™). Western blot analysis and immunoprecipitation were carried out with a monoclonal anti-hMLH1 antibody (clone: G168-728) and a monoclonal anti-hPMS2 antibody (clone: A16–4, BD Pharmingen). Immunofluorescence was performed with the Alexa Fluor 546 Goat Anti-Mouse IgG (H + L) secondary antibody (Thermo Scientific, Wilmington, DE) and mounting medium with DAPI for nuclear labeling (DAPI with Vectashield®, Vector Laboratories).

### Statistical analyses

The specificity and sensitivity of the Amsterdam criteria and the Bethesda guideline for identifying mutation carriers were calculated using two-by-two contingency tables with GraphPad Software. Associated 95% confidence intervals were calculated for each of the estimates of sensitivity and specificity.

Odds ratios (ORs) were used to estimate the relative risks for CRC and extra-colonic tumors in mutation carriers and noncarriers. ORs were generated from two-by-two tables, and statistical significance was assessed using Fisher’s exact test.

## Results

### Genetic analysis

A total of 116 Brazilian suspected LS patients were included in this study. From the 101 subjects of our previous study [12], 28 were mutation carriers (14 in *MLH1* and 14 in *MSH2*). Among the 73 *MLH1* and *MSH2*-negative patients, we evaluated point mutations in the *MSH6*, *PMS2* and *PMS1* genes. In addition, we included 15 new patients suspected of having LS for whom point mutations were evaluated for all five MMR genes (*MSH2*, *MLH1*, *MSH6*, *PMS2* and *PMS1*). Seven of these 15 patients carried pathogenic point mutations (five in *MSH2*, one in *MLH1* and one in *MSH6*).

From the complete cohort, 82 cases negative for *MLH1* and *MSH2* mutations were subjected to MLPA analysis to detect LGRs in the *MLH1*, *MSH2* and *MSH6* genes. We found copy number variations in the *MSH2* gene in six patients, representing 24% of all pathogenic mutations in this gene. This is the first description of three out of the six LGRs.

The analysis of the five MMR genes in the complete series of 116 patients revealed 45 carriers of pathogenic mutations (38.8%), including 25 (19 point mutations and six LGRs that generated copy number variations) in *MSH2* (55%), 15 point mutations in *MLH1* (33%), four point mutations in *MSH6* (9%) and one point mutation in *PMS2* (2%) (Table 1). We found no pathogenic mutations in the *PMS1* gene in this series. All five point mutations found in *MSH6* and *PMS2* are firstly described in the current study.

**Table 1:** Pathogenic mutations found in 116 LS patients.

ID	Gene	Loss of IHC	Alteration	Consequence	Exon	Cancer (age)	Inclusion criteria	Reference
2–10	*MSH2*	-	c.174dup	p.Lys59Glnfs*23	1	CC/SC (45/48)	BG	12
2–2	*MSH2*	-	c.187del	p.Val63fs*1	1	CC (21)	AC-I	12
170	*MSH2*	MSH2/MSH6	c.528_529del	p.Cys176*	03	SigC (61)	BG	Current study—Novel
154	*MSH2*	-	c.942+3A>T	-	i5	EC/CC/SC	AC-I	Current study -Insight
047	*MSH2*	MSH2/MSH6	c.942+3 A>T	-	i5	CCsinc (51/51)	AC-I	12
153	*MSH2*	MSH2/MSH6	c.1143_1144insA	p.Arg382Thrfs*7	07	EC/CC (53/53)	BG	Current study—Novel
043	*MSH2*	MSH2/MSH6	c.1444A>T	p.Arg482*	09	CC (53)	AC-I	12
150	*MSH2*	-	c.1444A>T	p.Arg482*	09	RC (32)	AC-I	Current study—Insight
008	*MSH2*	MSH2/MSH6	c.1447G>T	p.Glu483*	09	CCsin/RPC (27/44)	AC-I	12
010	*MSH2*	MSH2/MSH6	c.1667delT	p.Leu556*	11	CC (63)	AC-I	12
042	*MSH2*	MSH2/MSH6	c.1967_1970dup	p.Phe657Leufs*3	12	RC (15)	BG	12
028	*MSH2*	-	c.2131C>T	p.Arg711*	13	CC (62)	AC-I	12
165	*MSH2*	-	c.2145del	p. p.Asp716Thrfs*4	13	CC/EC/SC (28/33/43)	AC-I	Current study—Novel
020	*MSH2*	MSH2/MSH6	c.2152C>T	p.Gln718*	13	CCsin/EC (47/49)	AC-I	12
041	*MSH2*	MSH2/MSH6	c.2152C>T	p.Gln718*	13	CCsin (36)	AC-I	12
074	*MSH2*	MSH2/MSH6	c.2152C>T	p.Gln718*	13	EC (49)/CC (52/55)	AC-I	12
2–3	*MSH2*	-	c.2152C>T	p.Gln718*	13	CC/EC (41/52)	AC-I	12
2–9	*MSH2*	-	c.2152C>T	p.Gln718*	13	CC (29)	BG	12
017	*MSH2*	No	Exon 5 amplification	-	5	CC (68)	AC-I	Current study-Novel
006	*MSH2*	-	c. 2525_2526delAG	p.Glu842Valfs*3	15	CC (44)	AC-I	12
036	*MSH2*	-	EPCAM-MSH2 (exon1-4) deletion	-	1–4	CCsin/EC/GC (44/45/50)	AC-I	Current study—Novel
173	*MSH2*	-	Exon 6 deletion	-	6	CC (42)	Muir-Torre	Current study—Insight
003	*MSH2*	-	Exon 7 deletion	-	7	CC (29)	AC-I	Current study—Insight
024	*MSH2*	MSH2/MSH6	Exon 8 deletion	-	8	CCsin/SBC (43/51)	AC-I	Current study—Insight
072	*MSH2*	MSH2/MSH6	Exon 14 deletion	-	14	CC (51)	AC-I	Current study—Novel
156	*MLH1*	-	c.83C>T	p.Pro28Leu	01	CC (46)	AC-I	Current study—Insight
001	*MLH1*	MLH1/PMS2	c.545+3 A>G	-	06	CC/GC (40/42)	AC-I	12
2–1	*MLH1*	-	c.545+3A>G	-	06	CC (58)	AC-I	12
031	*MLH1*	PMS2	c.588+2T>A	-	07	CC/HC (43/50)	AC-I	12
021	*MLH1*	MLH1/PMS2	c.588+5G>C	-	07	CC/EC (36/47)	AC-I	12
2–8	*MLH1*	-	c.677G>A	p.Arg226Gln	08	RC/CC (31/44)	BG	12
023	*MLH1*	MLH1/PMS2	c.779T>G	p.Leu260Arg	09	CC (38)	BG	12
033	*MLH1*	PMS2	c.791-6_793del	-	10	CCsin (42)	AC-I	12
2–6	*MLH1*	-	c.1276C>T	p.Gln426*	12	CC (65)	BG	12
076	*MLH1*	-	c.1459C>T	p.Arg487*	13	CC (20)	BG	12
103	*MLH1*	-	c.1639_1643dup	p.Leu549Tyrfs*44	14	OC/EC/CC (37/37/45)	AC-I	12
058	*MLH1*	PMS2	c.1853delAinsTTCTT	p.Lys618Ilefs*4	16	CC (40)	AC-I	12
081	*MLH1*	PMS2	c.1975C>T	p.Arg659*	17	CCsin (28)	AC-I	12
099	*MLH1*	-	c.2041G>A	p.Ala681Thr	18	CC (47)	AC-I	12
022	*MLH1*	No	c.2224C>T	p.Gln742*	19	CC (32)	BG	12
152	*MSH6*	-	c.1483C>T	p.Arg495*	04	CC (59)	BG	Current study—Novel
050	*MSH6*	-	c.2379_2380del	p.Ala794Hisfs*9	04	EC/BC/PC (49/69/70)	AC-II	Current study—Novel
2–13	*MSH6*	-	c.3487G>T	p.Glu1163*	06	CC (39)	BG	Current study—Novel
032	*MSH6*	-	c.3974_3976del	p.Lys1325del	09	CC/LC (41/44)	BG	Current study—Novel
088	*PMS2*	-	c.1239dup	p.Asp414Argfs*45	11	CC (37)	AC-I	Current study—Novel

CC: Colon cancer; SC: Sebaceous carcinoma;SigC: Sigmoide adenocarcinoma; EC: Endometrial cancer; CCsin: Synchronic colon cancer; RC: Rectal cancer; RPC: Cancer of Renal Pelvis; SBC: Small Bowel cancer; GC: Gastric Cancer; HC: Hepatobiliary cancer; OC: Ovarian cancer, BC: Breast cancer; PC:Pancreatic cancer; LC: Lung cancer. AC-I: Amsterdam I; AC-II: Amsterdam II; BG: Bethesda guideline. The ref seq numbers for mutation description were: *MLH1* (NM_000249), *MSH2* (NM_000251.1), *MSH6* (NM_000179.1), *PMS1* (NM_000534.4) and *PMS2* (NM_000535.5); -: Not available

Interestingly, patient ID–036 carried a novel large deletion involving the *EPCAM-MSH2* genes, revealed by MLPA encompassing exon 3 of the *EPCAM* gene to exon 4 of *MSH2*. This patient displayed an aggressive phenotype with four primary tumors, including synchronous CRC at 44 years old, endometrial cancer at 45 and gastric cancer at the age of 50. She had a brother who also presented four primary tumors associated with the LS spectrum.

Patient ID–032, who carried a homozygous inframe deletion (c.3974_3976del; p.Lys1325del) in the *MSH6* gene, developed CRC at the age of 41 and had a sister who died of colangiocarcinoma at 44 years old (not assigned for genetic testing). In the first pedigree assessment, consanguineous marriage was not mentioned, but during the post-test informed consent, the patient revealed that his parents were cousins, which could potentially explain the homozygous status of the mutation. Interestingly, no other cases of colorectal cancers had been described for this family, suggesting that this mutation can be much less penetrant in the heterozygous status. However, this issue needs to be further addressed.

Loss of MMR proteins was assessed in tumors of 55 LS-suspected patients, including 21 out of 45 mutation carriers (Table 1) and 34 out of 71 noncarriers of mutations (noncarriers and VUS carriers) (S1 Table). For the group of carriers, six patients (6/21; 28%) presented conflicting results between IHC and gene mutation. In four patient, the tumors showed isolated loss of PMS2 but the mutations were found in *MLH1*. The other two cases revealed normal expression of the four proteins in one-exon amplification of *MSH2* and a premature stop codon in the last coding exon of *MLH1* gene (exon 19). All these unusual cases remains to be further investigated. For the group of noncarriers of pathogenic mutations, most of the cases showed no loss of MMR proteins, except two cases with loss of MLH1.

Point mutations in *KRAS* gene were found in 41.2% (7 out of 17 tumor samples) of colorectal tumors from patients with no identified MMR mutations. No mutation was identified either in *BRAF* (codon 600) or *NRAS* genes (codons 12, 13, 61 and 146) in this tumor set. MSI was found in 8% of tumor samples (2 out of 23). Interestingly, these two patients with MSI, also showed loss of MLH1 protein expression (abovementioned), including one MSI-H and one MSI-L, suggesting a possible mechanism of *MLH1* hypermethylation (S1 Table).

### Evaluation of VUS

Of the 71 patients without pathogenic MMR mutations, 10 were found to carry 13 different VUS, of which 11 are described here for the first time. *In silico* analysis of the VUS showed that 8 out of 13 were predicted to affect protein function and were consequently potentially pathogenic in at least one algorithm, whereas five were predicted to be potentially pathogenic in all three algorithms (S2 Table).

For the three most frequently mutated genes (*MSH2*, *MLH1* and *MSH6*), three variants were classified as potentially pathogenic in the three algorithms, two in *MSH2* (p.Ser153Cys and p.Leu173Arg) and one in *MLH1* (Leu676Pro), where the MLH1 Leu676Pro carrier was the only one to fulfill the Amsterdam criteria. Although the tumor profile of the Leu676Pro carrier did not showed MSI and IHC revealed normal nuclear staining of the four protein, we decided to further evaluate the impact of this novel variant on subcellular localization and MLH1-PMS2 dimerization, as suggested elsewhere [26].

Before initiating a functional analysis of the MLH1 Leu676Pro variant, we screened 280 DNA samples from healthy individuals with no history of cancer in the family to eliminate the possibility of this being a frequent polymorphism in our population. The Leu676Pro variant was not found in any of the healthy individuals. Additionally, to exclude the involvement of other cancer-predisposing genes in the MLH1 p.Leu676Pro carrier, we tested 14 additional cancer-predisposing genes using a gene panel containing 17 genes (*MLH1*, *MSH2*, *MSH6*, *ATM*, *BRCA1*, *BRCA2*, *CDKN2A*, *CHEK2*, *CTNNB1*, *ECAD*, *FANCJ*, *NBN*, *PALB2*, *PTEN*, *RAD50*, *RAD51* and *TP53*) by next-generation sequencing (NGS). No mutations were found in the 14 additional genes; the *MLH1* p.Leu676Pro variant, confirmed by NGS, was the only putative cancer-associated variant present in the patient (ID–039). This patient fulfilled the clinical Amsterdam criteria, with colon cancer at 43 years and breast cancer at the age of 50 (S1 Fig).

**Fig 1:**
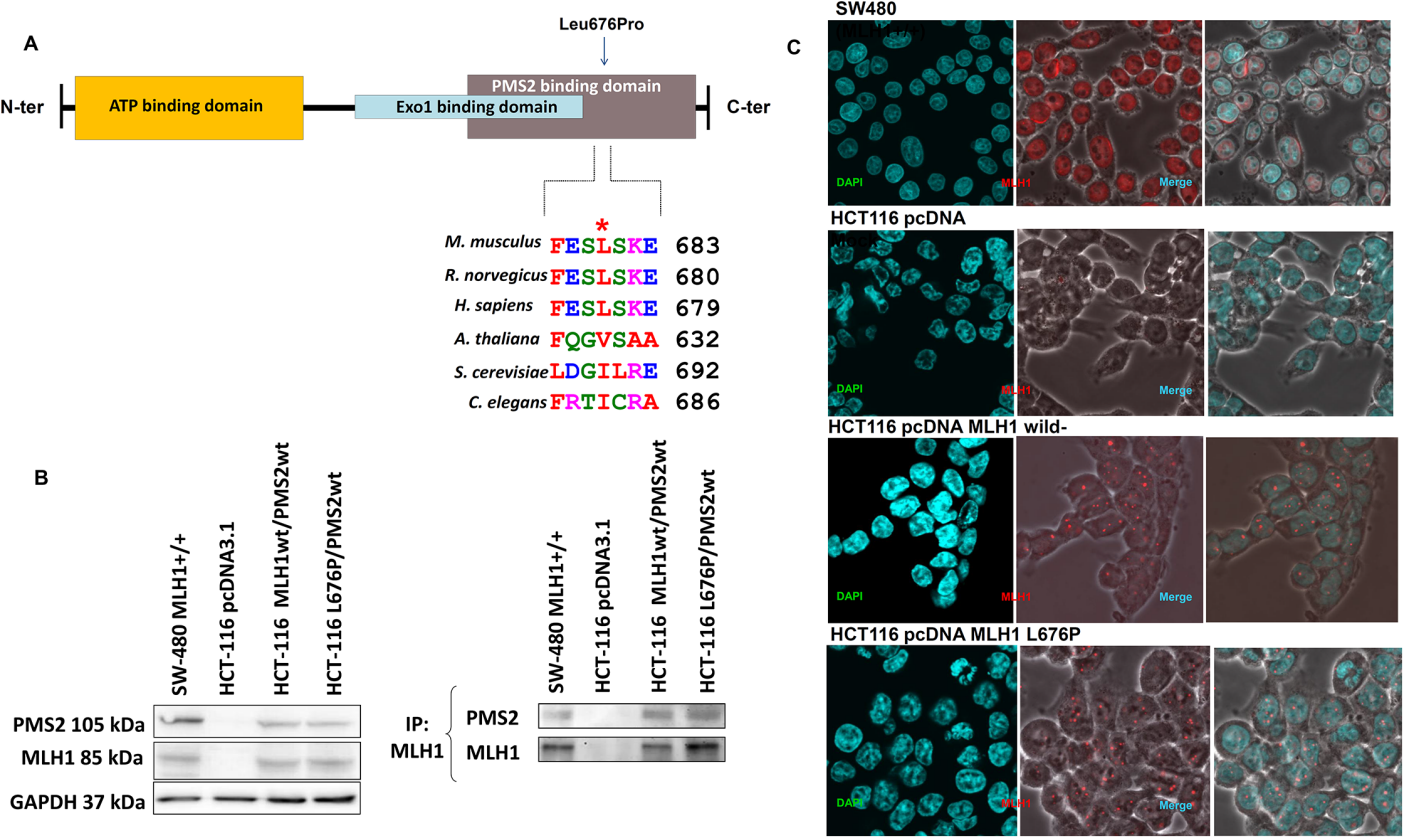
Functional analysis of the p.Leu676Pro missense MLH1 variant. (A) Functional domains of the MLH1 protein. The new missense alteration is located in the PMS2 interaction domain. The amino acid conservation across species is presented. (B) Western blot analysis for transient transfection and immunoprecipitation of MLH1 and PMS2. Immunoprecipitation analysis showed that the protein-protein interaction between the MLH1 Leu676Pro isoform and PMS2 was equivalent to that of wild-type MLH1 and PMS2, revealing no defective interaction. SW–480 was used as a positive control. (C) Immunofluorescence staining of wild-type MLH1 and MLH1 Leu676Pro. The mutant protein was correctly imported through the nuclear pore complex (NPC) and localized almost entirely in the nucleus, similar to the wild-type MLH1 protein.

The variant Leu676Pro is located in the COOH-terminal dimerization domain of MLH1, which is very important for the interaction with its dimerization partner PMS2 [27,28]. Thus, we investigated whether this amino acid change impairs either MLH1 subcellular localization or the MLH1-PMS2 interaction. The subcellular localization in HCT–116 cells expressing wild type or mutant MLH1 proteins was in both cases nuclear, showing that MLH1 was correctly imported and localized almost entirely in the nucleus in transfected cells (Fig 1). Additionally, western blot analysis indicated that the protein-protein interaction between the MLH1 p.Leu676Pro isoform and PMS2 was also very similar to that between wild-type MLH1 and PMS2, revealing no impairment in the interaction with its major partner.

### Assessment of mutation rate in LS-suspected patients fulfilling clinical criteria

Of the 116 families included, 49 fulfilled Amsterdam criteria I and II and 112 fulfilled the Bethesda guideline. The overall detection rate was 38.8%. According to the clinical criteria, the Amsterdam families had a positive detection rate of 61.2% (30/49), whereas patients fulfilling the Bethesda guideline had a rate of only 39.3% (44/112). The sensitivity and specificity of the Amsterdam criteria were 67% and 74%, respectively. As expected in our study, the Bethesda guideline was more sensitive (98%) but much less specific (4%). The Amsterdam criteria missed 15 (33%) mutation carriers, mainly due to the lack of three close relatives with CRC or LS-related tumors, and the Bethesda guideline missed 1 carrier who had CRC at the age of 65 (Table 2).

**Table 2:** Sensitivity and specificity of clinical criteria for detecting patients with pathogenic mutations in four MMR genes (*MLH1*, *MSH2*, *MSH6* and *PMS2*).

Clinical criteria	Families fulfilling criteria	Families positive for MMR mutations	Positive families missed by criteria	Sensitivity (95%CI)	Specificity (95%CI)
Amsterdam I and II	49	31	15	67% (52%-80%)	74% (62%-84%)
Bethesda	112	45	1	98% (88%-100%)	4% (1%-12%)

^CI: Confidence interval^

### Cancer occurrence in Lynch-suspected and confirmed families

Of the 116 families, we were able to evaluate the frequency of CRC and extra-colonic tumors in 2365 individuals. The number of individuals per family varied from 1 to 63, with a mean of 21, and a mean of four generations (1–5). The numbers of female and male relatives were 1136 and 1229, respectively.

Overall, we identified 325 CRC cases among the 2,365 individuals. Cancer in the right colon was found in 67.5% of the pathogenic mutation carrier group, whereas 78.6% of the non-carrier group had cancer in the left colon or rectum. Synchronous and metachronous tumors were described in 25% (10/40) of the mutation carriers in contrast with 2.9% (2/70) of the noncarriers (S3 Table).

**Fig 2:**
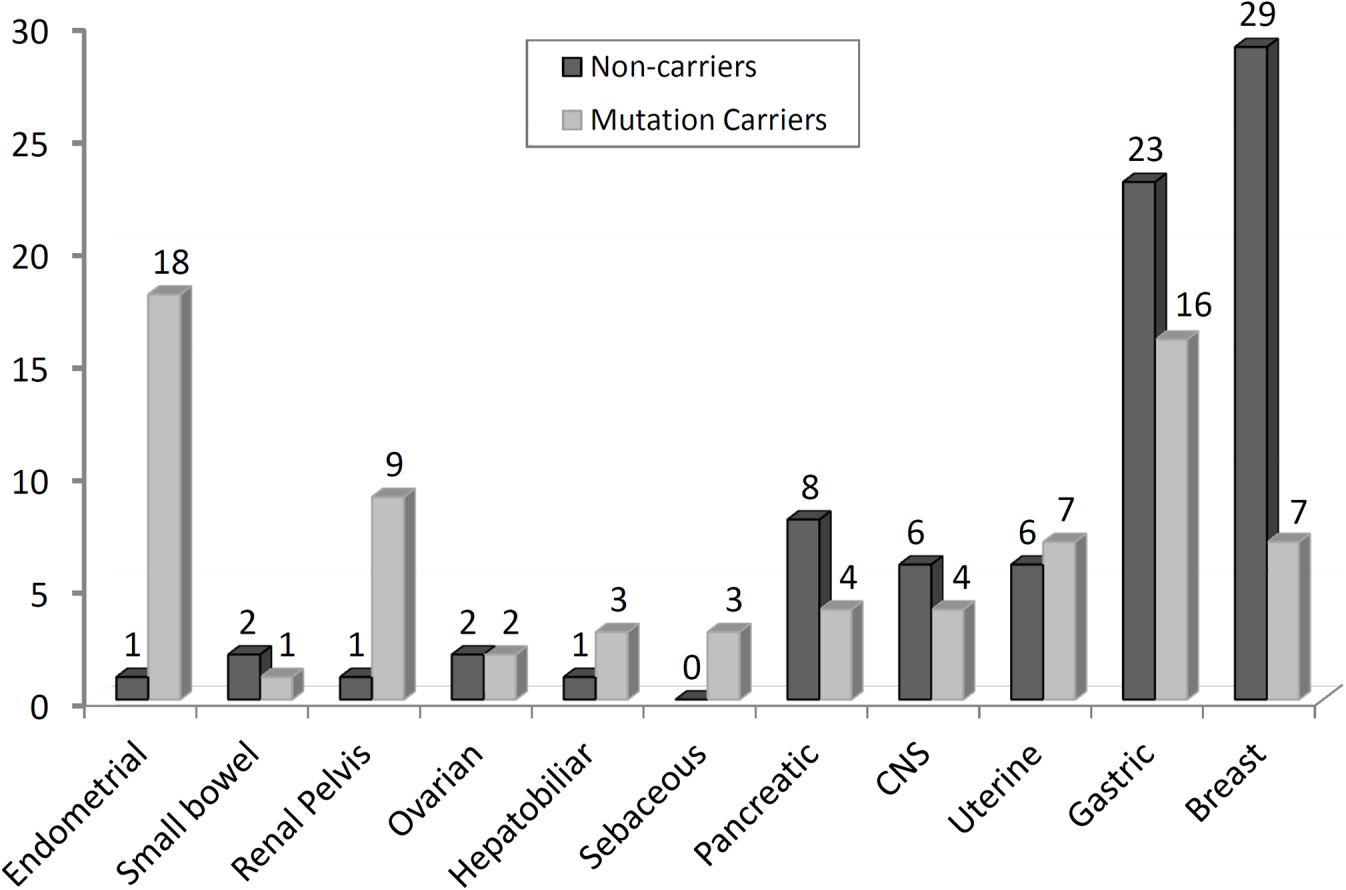
Frequency of extra-colonic tumors in 2365 family members of 116 LS patients.

Extra-colonic tumors were found 75 times in the 1,014 individuals from families with known pathogenic mutation. The most frequent extra-colonic manifestation in families of carriers was endometrial cancer (18 cases) followed by gastric cancer (16 cases. A slight majority of gastric cancer cases occurred among noncarriers of pathogenic MMR mutations (23 cases, 59%); however, 16 cases (41%) were detected among members of families with pathogenic mutations (9 in *MSH2* and 7 in *MLH1*); these subjects had gastric cancer with a low age at onset (mean, 49 years old). Breast cancer was the second most frequent tumor, with 7 cases (20%) in mutation carriers (3 in *MLH1* and 2 in *MSH2* and *MSH6*) and 29 cases (80%) in noncarriers (Table 3 and Fig 2). The mean age at breast cancer diagnosis was higher in mutation carriers than in noncarriers (59 versus 50, respectively).

**Table 3:** Number of CRC and extra-colonic tumors according to clinical criteria and mutation status.

	Amsterdam I & II		Bethesda		Odds Ratio (95%CI- carriers)
	MMR Carriers	MMR noncarriers	MMR carriers	MMR noncarriers	
Families (N = 116)	31	18	15	52	
Individuals (N = 2365)	911 (421 F/490 M)	477 (216 F/261 M)	103 (56 F/47 M)	874 (443 F/431 M)	
Colorectal cancer (N = 325)	159	62	21	88	1.6 (1.3–1.9)
Extra-colonic tumors (N = 163)					
Endometrial	16	0	2	1	25.8 (3.4–198)
Pelvis renal	9	1	0	0	12 (1.5–94)
Small-bowel	1	0	0	2	0.66 (0.06–7.3)
Uterine	6	4	1	2	1.6 (0.5–4.8)
Gastric	11	6	5	17	0.92 (0.5–1.7)
Hepatobiliar	2	0	1	1	4 (0.4–38)
Ovarian	2	0	0	2	1.38 (0.2–9.8)
Pancreas	4	1	0	7	0.66 (0.2–2.2)
CNS	4	3	0	3	0.88 (0.2–3.1)
Sebaceous	2	0	2	0	1.33 (0.18–9.4)
Breast	4	10	3	19	0.32 (0.1–0.7)

M:Male; F: Female

Endometrial cancer, as a component of LS tumors, was the third most frequent, with the great majority found in mutation carriers (18 out 19, 95%) (5 in *MLH1*, 8 in *MSH2* and 5 in *MSH6* carriers). Small-bowel and pelvis renal/ureter tumors, which are included in the original Amsterdam criteria II, were found in only one family harboring mutations and 9 cases in families of carriers, respectively. Regarding the renal pelvis/ureter tumors, eight of nine cases occurred in a single family carrying a *MSH2* mutation. The other tumors found in these carriers’ families included ovarian (two cases), hepatobiliar (three cases), sebaceous (four cases), uterine cervix (seven cases), pancreatic (four cases) and central nervous system (four cases) cancers.

## Discussion

This is the first comprehensive mutation analysis of the five mismatch repair genes, *MLH1*, *MSH2*, *MSH6*, *PMS2* and *PMS1*, in Brazilian families suspected of having Lynch syndrome. In our study, using direct sequencing of the five MMR genes and MLPA analysis for *MLH1*, *MSH2* and *MSH6* in a cohort of suspected LS patients, we were able to detect mutations in 38.8% (45/116) of the cases. Despite our increasing knowledge about the hereditary genetic basis of colorectal cancer, the identification of families with a molecular diagnosis of LS still remains problematic. The purpose of the existing clinical criteria (Amsterdam and Bethesda) is to identify those patients most likely to harbor pathogenic mutations in MMR genes. However, even with a comprehensive molecular screening of the five mismatch repair genes, a significant proportion of the patients still lack a molecular diagnosis.

Although the creation of clinical criteria has been important for the standardization of the LS diagnosis, the sensitivity and specificity of the clinical criteria for detecting LS patients are very far from the theoretical and ideal scenario, in which they should include in the LS-suspected group all patients with pathogenic MMR mutations (100% sensitivity) and no patients without a pathogenic mutation (100% specificity) in such genes. Because of our study design, which selected patients according to age of diagnosis and family history, it is believed that the results could be overrepresented, since patients with late age of cancer onset (>60 years) and no family history were not tested for mutations. Hence, the use of these criteria to guide genetic testing should be evaluated with great caution because the sensitivity and specificity, even that of the more restrictive criteria (Amsterdam), greatly reduces the molecular diagnosis of the syndrome [29]. In this sense, the use of immunohistochemistry (IHC) and microsatellite instability (MSI) testing, regardless the status of the clinical criteria, have been shown to be the most efficient method to guide genetic testing and have been widely used in major cancer centers. According to Vasen et al. (2013), the recommendations for identifying LS patients should be based on IHC of the four MMR proteins and MSI testing for all CRC cases diagnosed before the age of 70, regardless of clinical criteria [30].

At least eight extra-colonic malignancies are associated with LS, including cancers of the endometrium, ovary, stomach, ureter/renal pelvis, brain, small bowel, and hepatobiliary tract as well as sebaceous tumors. In our analysis, endometrial cancer was the most frequent extra-colonic manifestation in carriers of MMR mutations, which is the same as reported in other populations [31,32]. The apparent high incidence of renal pelvic tumors was attributable to a single family that carried an exon 7 deletion of the *MSH2* gene.

Despite the controversies about the involvement of breast cancer in LS [33], our data showed an inverse correlation between breast cancer and mutation carriers, where the majority of breast cancer cases were from families negative for MMR mutations. Moreover, the average age at breast cancer diagnosis in the negative families was 9 years younger (50 years) than in the MMR mutation-positive families (59 years) (no statistical significance), suggesting that genes other than MMR genes predispose to breast cancer in these families. However, the biggest challenge is to be able to differentiate phenocopies from the real MMR-associated cases [34]. Nevertheless, our findings suggest that the excess of breast cancer cases in families with colorectal cancer is more likely to be associated with another yet unknown syndrome rather than LS.

Germline mutations linked to LS are, in most cases, truncating mutations that cause loss of protein expression. However, missense mutations are very common and do not lead to loss of protein expression [35]. In our series, most patients with VUS had little or no family history of cancer. The analysis of the novel alterations was carried out by *in silico* programs that determine a specific score for mutations with high pathogenic potential. In this way, five missense variants were classified as likely to be pathogenic in all three algorithms (p.Ser153Cys and p.Leu173Arg in *MSH2*, p.Leu676Pro in *MLH1* and p.Ile679Thr and p.Ile755Thr in *PMS2*). Additionally, two variants were pathogenic in at least one algorithm, especially the variant p.Pro404Arg of *PMS2* gene, where histopathological analysis revealed loss of MLH1/PMS2 complex suggesting that this protein loss are more likely to be explained by this missense variant, and less likely due to two somatic mutations in either genes [36] or by a *MLH1* hypermethylation [37].

In this context, functional analyses have been widely used to investigate the possible impact on DNA MMR proteins [38,39], and although all VUS were candidates for functional analysis, in the current study we selected only the variant p.Leu676Pro in *MLH1*, which was found in a family fulfilling the Amsterdam criteria. To resolve the molecular defect of the variant, taking into consideration that this amino acid change occurred in the COOH-terminal dimerization domain of the MLH1 protein, we investigated whether this protein modification caused a deficiency in the MLH1-PMS2 interaction or in the nuclear translocation efficiency. Our analysis, based on functional assays, IHC (all expressed) and MSI status (MSS stable), suggested that the Leu676Pro variant is non-pathogenic. However, an MMR activity assay is mandatory to completely rule out a pathogenic effect.

Mutations in *MSH6* have been suggested to affect families with atypical LS, which manifests as a late onset of disease, lower incidence of colorectal cancer and a high incidence of endometrial cancer [15–20]. In this study, three out of four patients (ID–050, ID-2-13, ID–032) with pathogenic *MSH6* mutations had cancer early in life (Table 1), and patient LS–050 had endometrial cancer as her first tumor at the age of 49, which is a hallmark of *MSH6* mutation carriers [40]. According to Goodfellow et al. (2003), it is believed that 1.6% of all endometrial cases are related to germline mutations in the *MSH6* gene. However, our results showed that most of the patients carrying pathogenic mutations (and VUS) in *MSH6* did not fulfill the Amsterdam criteria, suggesting a less penetrant effect.

Until recently, attention has always been centered on heterozygous dominant mutations involving the MMR genes in LS. However, there are rare cases of biallelic mutations in these genes that gave rise to a new syndrome known as constitutive mismatch repair deficiency (CMMRD). In contrast to LS, individuals with biallelic mutations in MMR genes have no functional protein, and, generally, hematological and brain cancers develop in the first decade of life [41]. Interestingly, one of our patients (ID–032) showed a homozygous in-frame deletion in one aminoacid of exon 9 (p.Lys1325del) of the *MSH6* gene, which caused a deletion of a lysine at the carboxy-terminus of MSH6 protein. Although patients with biallelic mutations in *MSH6* have been reported with the CMMRD phenotype, including lymphomas, glioblastomas, astrocytomas and *café-au-lait* spots [42,43], our patient only had CRC at the age of 41 and a sister with colangiocarcinoma at the age of 44. One of the limitations of our data is that no further information of IHC staining and MSI status could be assessed to confirm the mutator phenotype of the lysine deletion found in MSH6. Nevertheless, we can hypothesize that this inframe deletion of unknown significance, if pathogenic, appears to have a low penetrance in the heterozygous case because no other relatives developed cancer. However, when inherited homozygously, the classical LS phenotype was acquired, rather than the CMMRD phenotype.

The detection of LS patients carrying mutations in *PMS2* is the most difficult task in LS diagnosis because mutations in *PMS2* lead to an attenuated phenotype, i.e., old age at onset, and, in most cases, the patients do not fulfill the stringent Amsterdam criteria [44]. Furthermore, the molecular diagnosis of PMS2-asssociated tumors is a great challenge because *PMS2* sequencing is not straightforward due to the existence of at least 13 regions of high homology throughout the gene, leaving its analysis limited to a few laboratories worldwide [45]. In our study, only one patient carried a truncating *PMS2* mutation (this patient also fulfilled the Amsterdam criteria), but a significant number of novel missense genetic variations were identified in this gene and should be further investigated.

The diagnosis of LS is often difficult due to the broad spectrum of tumors. The identification of families with genetic predispositions is very important for guiding genetic testing, management programs and increasing surveillance. Our analysis revealed that mutations were more frequent in *MSH2* than in *MLH1* in our population and that the frequencies of *MSH6*, *PMS2* and *PMS1* mutations were lower.

We have found that the sensitivity and specificity of the clinical criteria are below the rates recommended for use in genetic test guidance, corroborating the guideline proposed by Vasen et al. (2013) that every case of CRC before the age of 70 should be tested via MSI and IHC of the four MMR genes [27]. However, with the substantial advances in genomics and significant decrease in sequencing costs, genetic testing of the five MMR genes in at-risk patients by targeted gene panels may be the first and more effective measure for CRC patients in clinical practice.

## Supporting Information

S1 Fig Pedigree of Patient ID–36 submitted to functional analysis. This family fulfilled the Amsterdam criteria because of the three cases of CRC, one being a first-degree relative of the other two, at least one case occurring before the age of 50 years, and two successive generations affected.
(DOC)

S1 Table Analysis of MSI, IHC, Kras, Nras, Hras and Braf mutations in 43 out of 71 non mutation carriers and VUS carriers. CRC: Colorectal cancer; RC: Rectal cancer; EC: Endometrial cancer;MSS: Microsatellite stable; MSI-L: Low Microsatellite Instability
(DOC)

S2 Table *In silico* analysis of VUS detected in the five MMR genes. BC: Breast cancer; CC: Colon cancer; NA: Not Available; RC: Rectal cancer. AC-I: Amsterdam criteria I; AC-II: Amsterdam criteria II; BG: Bethesda guideline. Align GVGD: C0 (Less likely to interfere in protein function), C15, C25, C35, C45, C55, C65 (More likely to interfere in protein function); Polyphen: Variant benign, Possibly damaging and Probably damaging; SIFT: Variant tolerated (benign) or Affect protein function. LOVD /Insight class: class 1 (not pathogenic or of no clinical significance), class 2 (likely not pathogenic or of little clinical significance), class 3 (uncertain), class 4 (Likely pathogenic), Class 5 (Definitely pathogenic)
(DOC)

S3 Table Clinical features according to mutation status. Statistical significance when p≤ 0,05
(DOC)
