# Supplementary material for: Clinical and Molecular Characterization of Brazilian Patients Suspected to Have Lynch Syndrome
Source: PLoS One. 2015 Oct 5;10(10):e0139753. doi: 10.1371/journal.pone.0139753 (PMC4593564; doi:10.1371/journal.pone.0139753)
Supplement: S1 Fig — This family fulfilled the Amsterdam criteria because of the three cases of CRC, one being a first-degree relative of the other two, at least one case occurring before the age of 50 years, and two successive generations affected. (DOC) [file pone.0139753.s001.doc]

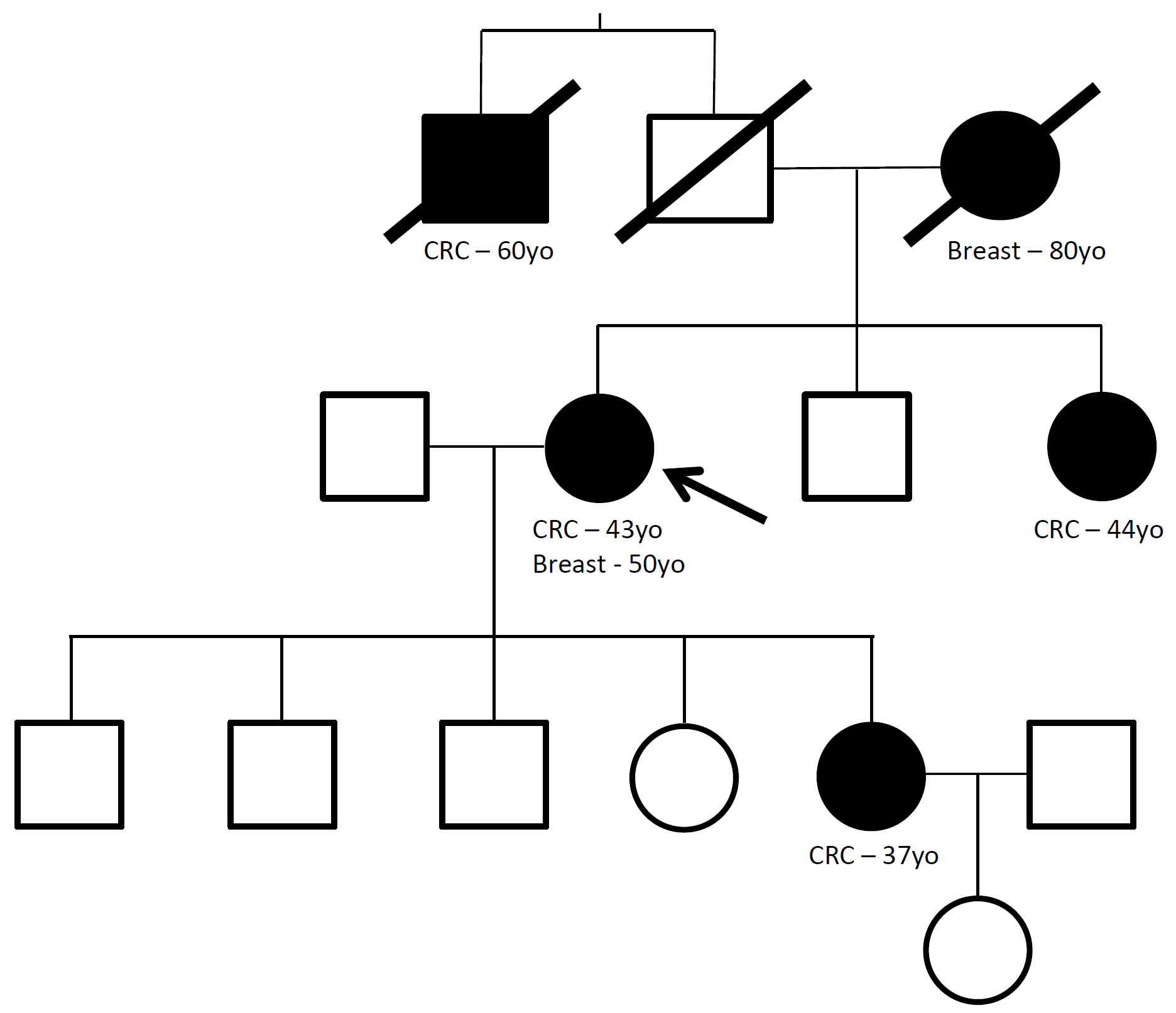


S2 Figure. Pedigree of Patient ID-36 submitted to functional analysis. This family fulfilled the Amsterdam criteria because of the three cases of CRC, one being a first-degree relative of the other two, at least one case occurring before the age of 50 years, and two successive generations affected.
