# Supplementary material for: Clinical and Molecular Characterization of Brazilian Patients Suspected to Have Lynch Syndrome
Source: PLoS One. 2015 Oct 5;10(10):e0139753. doi: 10.1371/journal.pone.0139753 (PMC4593564; doi:10.1371/journal.pone.0139753)
Supplement: S1 Table — CRC: Colorectal cancer; RC: Rectal cancer; EC: Endometrial cancer;MSS: Microsatellite stable; MSI-L: Low Microsatellite Instability (DOC) [file pone.0139753.s002.doc]

**S1 Table. Analysis of MSI, IHC, Kras, Nras, Hras and Braf mutations in 43 out of 71 non mutation carriers and VUS carriers.**

| ID | Inclusion criteria | Cancer (age) | Kras (frequency >5%) | Nras (frequency >5%) | Braf status | Microsatellite status | Loss of protein |
| --- | --- | --- | --- | --- | --- | --- | --- |
| 018 | AC-I | CC (49) | - | - | - | MSS | No |
| 019 | BG | CC (49) | Wild type | Wild type | Wild type | MSS | No |
| 029 | BG | EC (39) | Wild type | Wild type | Wild type | MSS | - |
| 040 | AC-I | CC (33) | Wild type | Wild type | Wild type | MSS | No |
| 045 | AC-I | CC (71) | - | - | - | MSS | No |
| 055 | BG | RC ( 39) | p.Gly12Asp | Wild type | Wild type | MSS | No |
| 056 | BG | CC (48) | p.Gly12Asp | Wild type | Wild type | MSI-H | MLH1 |
| 059 | BG | RC ( 48) | Wild type | Wild type | Wild type | MSS | No |
| 060 | AC-I | CC (57) | p.Gly13Asp | Wild type | Wild type | MSS | No |
| 061 | BG | CC (39) | - | - | - | MSS | No |
| 063 | AC-I | CC (56) | Wild type | Wild type | Wild type | MSI-L (1/5) | MLH1/PMS2 |
| 066 | BG | CC (52) | - | - | - | MSS | NA |
| 067 | BG | CC (41) | - | - | - | MSS | No |
| 070 | BG | CC (44) | - | - | - | MSS | No |
| 071 | BG | CC (36) | p.Ala146Thr | Wild type | Wild type | MSS | No |
| 077 | BG | CC (48) | Wild type | Wild type | Wild type | MSS | - |
| 085 | BG | CC (51) | p.Gly12Ser | Wild type | Wild type | MSS | No |
| 092 | BG | CC (35) | p.Gly12Val | Wild type | Wild type | MSS | - |
| 094 | BG | CC (41) | Wild type | Wild type | Wild type | MSS | - |
| 096 | BG | RC (31) | Wild type | Wild type | Wild type | MSS | - |
| 163 | BG | CC (48) | p.Gly12Val | Wild type | Wild type | MSS | - |
| 166 | BG | CC (42) | Wild type | Wild type | Wild type | MSS | - |
| 167 | BG | CC (45) | Wild type | Wild type | Wild type | MSS | - |
| 016 | AC-I | CC (49) BREAST (51) | - | - | - | - | No |
| 025 | BG | CC(53) | - | - | - | - | No |
| 030 | BG | RC (63) | - | - | - | - | No |
| 049 | BG | CC (51) | - | - | - | - | No |
| 053 | BG | CC (50) | - | - | - | - | No |
| 054 | BG | CC (36) | - | - | - | - | No |
| 002 | BG | CC (31) | - | - | - | - | No |
| 062 | BG | CC (39) | - | - | - | - | No |
| 064 | BG | CC (65) | - | - | - | - | No |
| 065 | BG | CC (34) | - | - | - | - | No |
| 068 | BG | CC (43) | - | - | - | - | No |
| 069 | BG | CC (46 | - | - | - | - | No |
| 073 | AC-I | CC (25) | - | - | - | - | No |
| 075 | BG | CC (40) | - | - | - | - | No |
| 078 | BG | RC (24) | - | - | - | - | No |
| 080 | BG | RC (31) | - | - | - | - | No |
| 082 | BG | CC (72) | - | - | - | - | No |
| 091 | BG | CC (38) | - | - | - | - | No |
| 100 | BG | CC (51) | - | - | - | - | No |
| 155 | AC-I | CC (46) | - | - | - | - | No |
|  |  |  |  |  |  |  |  |
|  |  |  |  |  |  |  |  |

AC-I: Amsterdam criteria; BG: Bethesda guideline; CC: Colon cancer; RC: Rectal cancer; EC: Endometrial cancer;MSS: Microsatellite stable; MSI-L: Low Microsatellite Instability; NA: Not available
