# Supplementary material for: Clinical and Molecular Characterization of Brazilian Patients Suspected to Have Lynch Syndrome
Source: PLoS One. 2015 Oct 5;10(10):e0139753. doi: 10.1371/journal.pone.0139753 (PMC4593564; doi:10.1371/journal.pone.0139753)
Supplement: S2 Table — BC: Breast cancer; CC: Colon cancer; NA: Not Available; RC: Rectal cancer. AC-I: Amsterdam criteria I; AC-II: Amsterdam criteria II; BG: Bethesda guideline. Align GVGD: C0 (Less likely to interfere in protein function), C15, C25, C35, C45, C55, C65 (More likely to interfere in protein function); Polyphen: Variant benign, Possibly damaging and Probably damaging; SIFT: Variant tolerated (benign) or Affect protein function. LOVD /Insight class: class 1 (not pathogenic or of no clinical significance), class 2 (likely not pathogenic or of little clinical significance), class 3 (uncertain), class 4 (Likely pathogenic), Class 5 (Definitely pathogenic) (DOC) [file pone.0139753.s003.doc]

**S2 Table: *In silico*** analysis of VUS detected in the five MMR genes

| **ID** | **Alteration** | **Consequence** | **Loss of IHC** | **Cancer (age)** | **Clinical criteria** | **SIFT** | **Polyphen-2** | **Align GVGD** | **LOVD Insight class** | **Reference** |
| --- | --- | --- | --- | --- | --- | --- | --- | --- | --- | --- |
| ***MLH1*** |  |  |  |  |  |  |  |  |  |  |
| 039 | c.2027T>C | p.Leu676Pro | MMR+ | BC, CC (48/56) | AC-I | Affect Protein | Probably damaging | C15 | Class 3 | Valentin et al. 2011 |
| ***MSH2*** |  |  |  |  |  |  |  |  |  |  |
| 162 | c.458C>G | p.Ser153Cys | NA | NA | BG | Affect Protein | Possibly damaging | C15 | NA | Current study |
| 172 | c.518T>G | p.Leu173Arg | NA | NA | BG | Affect Protein | Probably damaging | C35 | Class 3 | LOVD Database |
| ***MSH6*** |  |  |  |  |  |  |  |  |  |  |
| 071 | c.124C>T | p.Pro42Leu | MMR + | CC (36) | BG | Tolerated | Benign | C0 | Class 3 | Current study |
| 071 | c.1338A>T | p.Glu446Asp | MMR + | CC (36) | BG | Affect Protein | Benign | C35 | Class 3 | Current study |
| 071 | c.1932G>C | p.Arg644Ser | MMR + | CC (36) | BG | Tolerated | Benign | C0 | Class 3 | Current study |
| ***PMS1*** |  |  |  |  |  |  |  |  |  |  |
| 026 | c.1625A>G | p. Met539Val | NA | RC (34) | BG | Tolerated | Benign | NA | NA | Current study |
| 098 | c.985C>G | p.Leu329Val | NA | CC (38) | BG | Tolerated | Benign | NA | NA | Current study |
| 2-7 | c.2417C>G | p.Thr806Ser | NA | CC (37) | BG | Tolerated | Benign | NA | NA | Current study |
| ***PMS2*** |  |  |  |  |  |  |  |  |  |  |
| 2-7 | c.1144G>C | p.Gly382Arg | NA | CC (37) | BG | Tolerated | Probably damaging | C0 | NA | Current study |
| 053 | c.2036T>C | p.Ile679Thr | MMR+ | CC (50) | BG | Affect Protein | Possibly damaging | C25 | NA | Current study |
| 062 | c.2264T>C | p.Ile755Thr | MMR + | CC (39) | BG | Affect Protein | Probably damaging | C35 | NA | Current study |
| 063 | c.1211C>G | p.Pro404Arg | MLH1-PMS2 | CC (56) | AC-I | Tolerated | Possibly damaging | C0 | NA | Current study |

BC: Breast cancer; CC: Colon cancer; NA: Not Available; RC: Rectal cancer. AC-I: Amsterdam criteria I; AC-II: Amsterdam criteria II; BG: Bethesda guideline. Align GVGD: C0 (Less likely to interfere in protein function), C15, C25, C35, C45, C55, C65 (More likely to interfere in protein function); Polyphen: Variant benign, Possibly damaging and Probably damaging; SIFT: Variant tolerated (benign) or Affect protein function. LOVD /Insight class: class 1 (not pathogenic or of no clinical significance), class 2 (likely not pathogenic or of little clinical significance), class 3 (uncertain), class 4 (Likely pathogenic), Class 5 (Definitely pathogenic)
