# Supplementary material for: Clinical and Molecular Characterization of Brazilian Patients Suspected to Have Lynch Syndrome
Source: PLoS One. 2015 Oct 5;10(10):e0139753. doi: 10.1371/journal.pone.0139753 (PMC4593564; doi:10.1371/journal.pone.0139753)
Supplement: S3 Table — Statistical significance when p≤ 0,05 (DOC) [file pone.0139753.s004.doc]

**S3 Table. Clinical features according to mutation status**

| Features | Non-carriers | Carriers | p value |
| --- | --- | --- | --- |
| **Tumor localization** |  |  |  |
| Ascending colon | 15 (21.4%) | 27 (67.5%) |  |
| Descending colon and rectal | 55 (78.6%) | 13 (22.5%) | 0.001 |
| **Histologic type** |  |  |  |
| Tubular | 56 (80%) | 27 (67.5%) |  |
| Mucinous | 14 (20%) | 13 (32.5%) | 0.1 |
| **Synchronous or metachronous** |  |  |  |
| Yes | 2 (2.9%) | 10 (25%) |  |
| No | 68 (97.1%) | 30 (75%) | 0.001 |
| **Perineural invasion** |  |  |  |
| Yes | 29 (76.3%) | 17 (100%) | 0.02 |
| No | 9 (23.7%) | 0 |  |

Statistical significance when p≤ 0,05
